# Mitochondrial aerobic respiration is activated during hair follicle stem cell differentiation, and its dysfunction retards hair regeneration

**DOI:** 10.7717/peerj.1821

**Published:** 2016-05-03

**Authors:** Yan Tang, Binping Luo, Zhili Deng, Ben Wang, Fangfen Liu, Jinmao Li, Wei Shi, Hongfu Xie, Xingwang Hu, Ji Li

**Affiliations:** 1 Department of Dermatology, Xiangya Hospital, Central South University, Changsha, Hunan, China; 2 Department of Dermatology, The Third Xiangya Hospital, Central South University, Changsha, Hunan, China; 3 Department of Infectious Diseases and Hunan Key Laboratory of Viral Hepatitis, Xiangya Hospital, Central South University, Changsha, Hunan, China

**Keywords:** Hair follicle stem cell, Mitochondria, Differentiation, Hair regeneration, Redox balance

## Abstract

**Background.** Emerging research revealed the essential role of mitochondria in regulating stem/progenitor cell differentiation of neural progenitor cells, mesenchymal stem cells and other stem cells through reactive oxygen species (ROS), Notch or other signaling pathway. Inhibition of mitochondrial protein synthesis results in hair loss upon injury. However, alteration of mitochondrial morphology and metabolic function during hair follicle stem cells (HFSCs) differentiation and how they affect hair regeneration has not been elaborated upon.

**Methods.** We compared the difference in mitochondrial morphology and activity between telogen bulge cells and anagen matrix cells. Expression levels of mitochondrial ROS and superoxide dismutase 2 (SOD2) were measured to evaluate redox balance. In addition, the level of pyruvate dehydrogenase kinase (PDK) and pyruvate dehydrogenase (PDH) were estimated to present the change in energetic metabolism during differentiation. To explore the effect of the mitochondrial metabolism on regulating hair regeneration, hair growth was observed after application of a mitochondrial respiratory inhibitor upon hair plucking.

**Results.** During HFSCs differentiation, mitochondria became elongated with more abundant organized cristae and showed higher activity in differentiated cells. SOD2 was enhanced for redox balance with relatively stable ROS levels in differentiated cells. PDK increased in HFSCs while differentiated cells showed enhanced PDH, indicating that respiration switched from glycolysis to oxidative phosphorylation during differentiation. Inhibiting mitochondrial respiration in differentiated hair follicle cells upon hair plucking repressed hair regeneration *in vivo*.

**Conclusions.** Upon HFSCs differentiation, mitochondria are elongated with more abundant cristae and show higher activity, accompanying with activated aerobic respiration in differentiated cells for higher energy supply. Also, dysfunction of mitochondrial respiration delays hair regeneration upon injury.

## Introduction

Hair follicle (HF) is a cystic tissue surrounding the hair root, controlling hair growth. It consists of two parts: an epithelial part (hair matrix and outer root sheath) and a dermal part (dermal papilla and connective tissue sheath). The hair follicle goes through cycles of anagen phase (growth), catagen phase (degeneration) and telogen phase (rest) (Stenn & Paus, 2001). Hair follicle stem cells (HFSCs) have a slow cell cycle and play a crucial role in hair growth, regeneration of epidermis and sebaceous glands, and skin reparation after injury (Varum et al., 2011). In the late telogen phase, hair follicle bulge stem cells differentiate into matrix cells upon stimulation, to re-enter the anagen phase. While in the catagen phase, proliferation and differentiation of hair follicle cells gradually attenuates, leaving with HFSCs and a dormant hair germ, re-entering the telogen phase (Lien et al., 2011).

Survival of stem cells such as hematopoietic stem cells (HSCs), embryonic stem cells (ESCs) and induced pluripotent stem cells (iPSCs) depend mostly on anaerobic metabolism rather than on aerobic metabolism, while terminally differentiated cells adopt themselves with aerobic respiration (Hsu & Cheng-Kui, 2013; Jang et al., 2015; Kondoh et al., 2007; Teslaa, Teitell & Michael, 2015; Varum et al., 2011). As an essential organelle for anaerobic respiration, mitochondria attracted more research attention to its morphology and function during stem cell differentiation. Mitochondria show less mass in ESCs than that in differentiated cells, with a reduced oxygen consumption rate and less ROS (Cho et al., 2006; Choi et al., 2015; Lyu et al., 2008). Effective control of mitochondrial morphology and function is critical for the maintenance of energy production and the prevention of oxidative stress-induced damage (Parker, Acsadi & Brenner, 2009). Besides, mitochondria play an essential role in determining hair cell differentiation and proliferation upon injury though regulating energy metabolism (Armstrong et al., 2010; Hamanaka & Chandel, 2013). In addition, ROS inhibit stem cell differentiation and proliferation through redox signaling pathway (Ghaffari, 2008; Naka et al., 2008). Therefore, to counteract the adverse effect of ROS, the level of enzymes such as SOD2 is subsequently up-regulated.

It has been reported that inhibition of mitochondrial protein synthesis can increase area of hair loss by 30–80% (Hyde & Rubel, 1995). But the mechanism behind this phenomenon has not been fully illustrated. Recently, increasing studies have revealed the significance of mitochondria in regulating stem/progenitor cell differentiation and cell proliferation of keratinocytes, neural progenitor cells (NPCs) and bone marrow derived mesenchymal stem cells (bmMSCs) (Hamanaka & Chandel, 2013; Kasahara & Scorrano, 2014; Kloepper et al., 2015). However, the changes in mitochondrial morphology and function, especially energy metabolism during HFSC differentiation are not well established.

This paper revealed the alterations in mitochondrial morphology and activity during HFSCs differentiation and the effect of mitochondrial function in regulating hair regeneration. A more sophisticated mitochondrial ultrastructure showing elongation with abundant organized cristae and an increased mitochondrial activity were discovered in hair follicle cells upon differentiation. The level of SOD2 was elevated to maintain the redox homeostasis during differentiation. Furthermore, inhibiting mitochondrial aerobic respiration repressed plucking-induced hair regeneration.

## Materials and Methods

### Experimental animals

Eight-week-old C57BL/6 mice were used in all experiments except the old mice group (aged two-year old). All experiments were repeated at least three times with 3–5 mice per experiment. All animals received humane care, maintained in seperate cages with general rodent diet between 22 °C–24 °C.

The study was approved by the Ethics Committee of the Center, Scientific Research Center with Animal Models, Xiangya Hospital, Central South University (No: 2011-01-05). All procedures on animals followed the guidelines for humane treatment set by the Ethics Committee of the Center, Scientific Research Center with Animal Models, Xiangya Hospital, Central South University.

### Preparation of tissue samples

Ketamine (80 mg/kg per mice) and Xylazine (5 mg/kg per mice) were injected i.p. before tissue preparation. After anesthesia, the skin samples with different phases of hairs were obtained from the back of mice after anesthesia, and the wound was sewed afterwards. Then the skin samples were incubated in 0.25% solution of Dispase (Dispase I, Sigma-Aldrich Co. LLC) in Hanks’ balanced salt solution (HBSS, Life technologies, Thermo fisher Scientific Inc.,Grand Island, NY) at 4 °C overnight. Based on previous research, hair follicles represent grey or black in anagen phase, while showing pink with no pigment during telogen phase (Maksim & Plikus, 2009). And the epidermis with telogen hair follicles or anagen hair follicles was separated with forceps under a binocular light microscope according to skin color and morphology.

### MitoTracker

The telogen hair follicles with epidermis and the whole anagen hair follicles were incubated in 50 nM MitoTracker media (MitoTracker^®^ Red CMXRos; Life Technologies, Thermo Fisher Scientific Inc., Waltham, MA, USA) for 30 min at 37 °C. After incubation and washing, the tissues were incubated with 3 uM DAPI (DAPI, 4′, 6-Diamidino-2-Phenylindole, Dilactate, Life technologies, Thermo fisher Scientific Inc., Waltham, MA, USA) in PBS for 10 min at room temperature. Then the samples were mounted on the glass slide covered with glycerol and observed with a con-focal microscope.

### Transmission electron microscope (TEM)

Immediately after removal of the mouse skin, tissues were sliced into small pieces (1 mm^3^) and fixed in 3% buffered glutaraldehyde (Glutaraldehyde 25% solution; Sigma-Aldrich Co. LLC) for 4 h at 4 °C. Tissue specimens were then fixed in 1% osmium tetroxide (OsO^4^, ReagentPlus^®^ , 99.8%; Sigma-Aldrich Co. LLC) for 90 min. Fixed tissue was dehydrated using ascending grades of ethanol and transferred into the resin via propylene oxide. After impregnation with pure resin, specimens were embedded in the same resin mixture. Ultra-thin sections of silver shades (60–70 nm) were cut using an ultra-microtome (Leica Rotary Microtome RM2255; Leica, Wetzlar, Germany) equipped with a diamond knife; sections were then placed on copper grids and stained with uranyl acetate (20 min) and lead citrate (5 min). Stained sections were observed with a TEM (JEOL JEM-1011) operating at 80 kV.

### Detection of ROS

The telogen hair follicles with epidermis and the whole anagen hair follicles were washed with PBS and treated with 10 µM DCFDA (29,79-dichlorofluorescein diacetate, DCFDA-Cellular Reactive Oxygen Species Detection Assay Kit; AbcamInc., Cambridge, MA, USA) in DMEM (Dulbecco’s Modified Eagle Medium, Life Technologies, Thermo Fisher Scientific Inc.) for 20 min at 37 °C in the dark. The samples were washed with PBS 4 times and then mounted on a glass slide covered with glycerol and observed with a confocal microscopy.

### Immunohistochemical Staining and Immunofluorescence staining

First, the skin samples were fixed in 4% Paraformaldehyde overnight at 4 °C and embedded with paraffin. After deparaffin and hydration, the samples sections were treated in boiling 0.01 M Tri-Sodium Citrate buffer (pH 6.0) for 20 min in water bath for antigen retrieval. And the samples were then incubated in 3% H_2_O_2_ at room temperature (22 °C –24 °C) for 10 min to quench endogenous peroxidase. Immunostaining procedure was carried out according to the manufacturer’s instructions for the M.O.M kit (Cat No. PK-220; Vector Laboratories Inc., Burlingame, CA, USA). The samples were incubated with primary antibodies for rabbit anti-SOD1 (1:200, Abcam Inc., Cambridge, MA) or mouse anti-SOD2 (1:200; Abcam Inc., Cambridge, MA, USA) overnight at 4 °C. The DAB substrate kit (Abcam Inc., Cambridge, MA, USA) was used for color development.

Early anagen hair follicles with epidermis were fixed in 100% methanol for 1 h. Fixed samples were treated with 0.5% triton X-100 for 15 min at room temperature (22 °C –24 °C) and blocked with 5% bovine serum albumin for 1 h at 37 °C. After rinsing with PBS, the samples were incubated at 4 °C overnight with PDK or PDH antibodies (1:200; Santa Cruz Biotechnology Inc., Dallas, TX, USA), and then incubated in antibodies against K15 and Ki67, respectively, at 37 °C for 1 h. Samples were rinsed with PBS four times (5 min each time) and incubated in the dark for 1 h at 37 °C with two appropriate fluorescence-labeled secondary antibodies. After rinsing with PBS 4 times (5 min each time), the samples were incubated with 3 µM DAPI in PBS and then were mounted on glass slides covered with glycerol and observed with con-focal microscopy.

Length of mitochondria was measured through Image pro plus 6.0. Integrated optical density (IOD) and area of figures were evaluated by Image pro plus 6.0, and mean density was calculated as: Mean density = IOD/area. A two-tailed student’s t test was used for comparison.

### Drug preparation

Antimycin A stock (Sigma-Aldrich Co. LLC) was made by dissolving in DMSO, and the working solution was made by diluting the stock solution with DMEM to a final concentration of 10 µM prior to use.

### Hair regeneration *in vivo*

Synchronous anagen was induced by depilation in the back skin of mice with all dorsal skin HFs in telogen stage of the hair cycle as described by Muller-Rover et al. (Porter, 2003). After HFs switched from anagen to telogen, we injected 100 µl antimycin A (experiment group) and DMSO (control group) intracutaneously on respective side of the mouse back for 10 days and 200 hairs were plucked at the drug treated sites at the 3rd day of treatment (Day 3). Mice were separated in different cages (1 mice per cage) and under close observation everyday. Pictures were taken on the studied location on mouse back every day and the time when the hair grows out was recorded.

### Statistical analysis

Non-parametric Mann Whitney test was performed for comparison through GraphPad Prism 6.0 software. A value of *P* < 0.05 was considered statistically significant.

## Results

### Change of mitochondrial ultrastructure during hair follicle bulge cells differentiation

During telogen phase, the inferior part of the hair follicle consists of mostly bulge stem cells and secondary hair germ (Mayumi Ito, Kenji & Kazuto, 2004). The bulge stem cells differentiate into proliferating matrix cells, entering the anagen phase (Wilson et al., 1994; Hideo Oshima et al., 2001). Hence in this paper, telogen phase hair follicle bulge cells (abbreviated as telogen bulge cells) and anagen phase proliferating hair follicle matrix cells (abbreviated as anagen matrix cells) were used as representative of HFSCs and differentiated HF cells respectively to detect the change in mitochondrial morphology and function during HFSCs differentiation.

**Figure 1:**
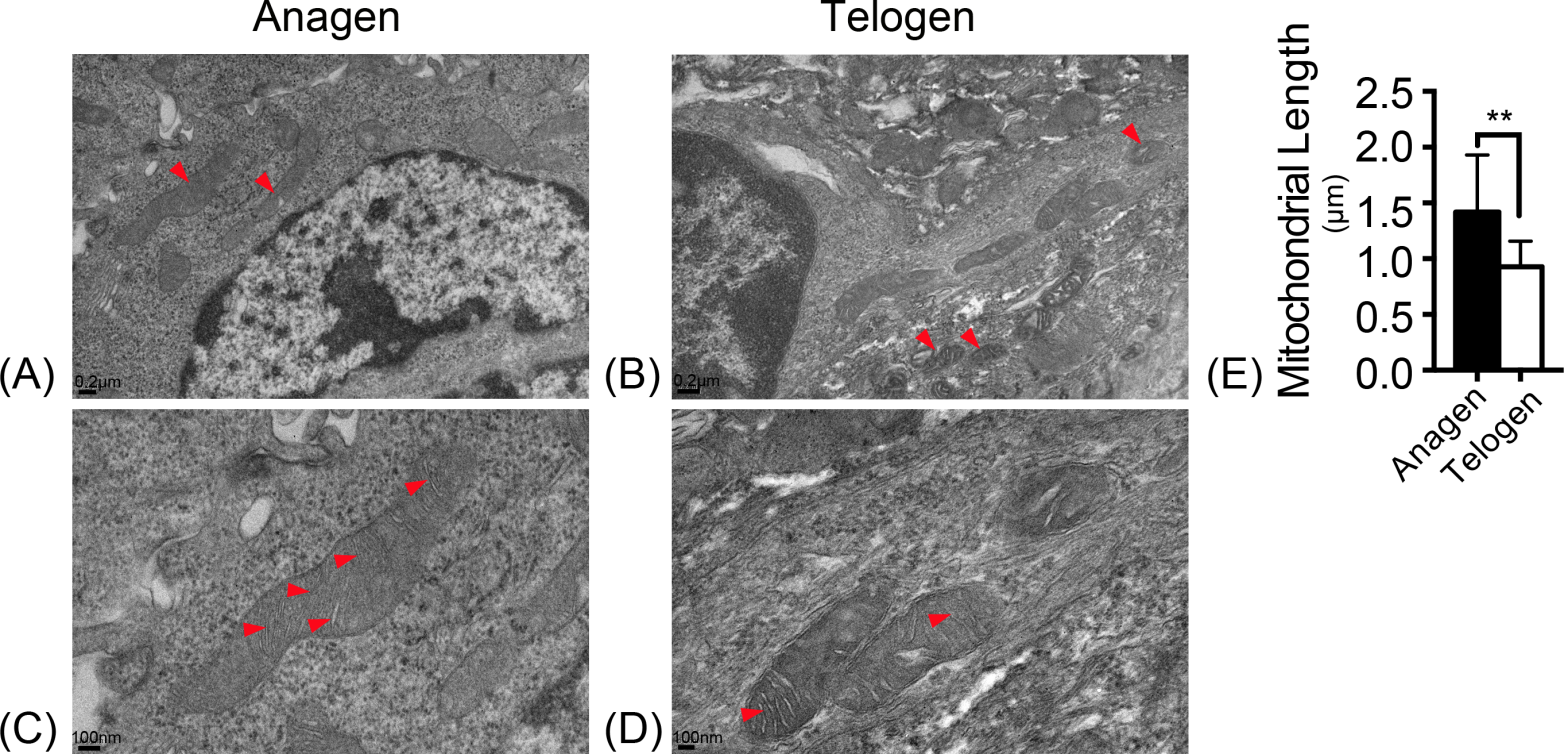
Mitochondria are elongated with abundant cristae in anagen matrix cells. Magnifications of (A) 10×, and (C) 20× of mitochondria ultrastructure in anagen phase differentiated hair follicle matrix cells. More elongated mitochondria shown in (A) anagen phase, while more discrete, spherical mitochondria shown in (B) telogen phase bulge cells. (Marked by red arrows) Magnifications of (B) 10×, and (D) 20×of mitochondria ultrastructure in telogen phase bulge cells. Mitochondria in (C) anagen phase matrix cells showed more abundant cristae than in (D) telogen phase bulge cells. (Marked by red arrows) (E) The lengths of mitochondria measured in anagen matrix cells were significantly shorter than in telogen bulge cells (^∗∗^, *P* < 0.01). Data show a complication of 3 experiments (*n* = 4 mice per group, with two 5 mm × 5 mm sections per mice).

Mitochondrial morphology was observed with electron microscopy. In the ultrastructure, more mitochondria were observed in anagen matrix cells (Fig. 1A) and they were elongated with organized cristae (Fig. 1C). However, mitochondria were discrete and spherical in telogen bulge cells (Fig. 1B) with less cristae (Fig. 1D). And the average length of mitochondria in anagen matrix cells was significantly increased than that in telogen bulge cells (Fig. 1E). Accordingly, the mitochondria became more sophisticated in ultrastructure after differentiation, implying that differentiated matrix cells have a higher energetic potential.

### Alteration of mitochondrial activity in HFSCs differentiation

It was previously observed that iPSCs typically have glycolytic energy production at pluripotent phase, whereas oxidative phosphorylation is essential during cell proliferation and differentiation. In addition to the reduction of energy metabolism, iPSCs also have less mitochondria and lower mitochondrial activity than that in differentiated cells. The alteration of the mitochondrial morphology and function is a crucial marker of iPSCs differentiation (Varum et al., 2011). However, the change of mitochondrial activity during HFSCs differentiation has not been elucidated.

**Figure 2:**
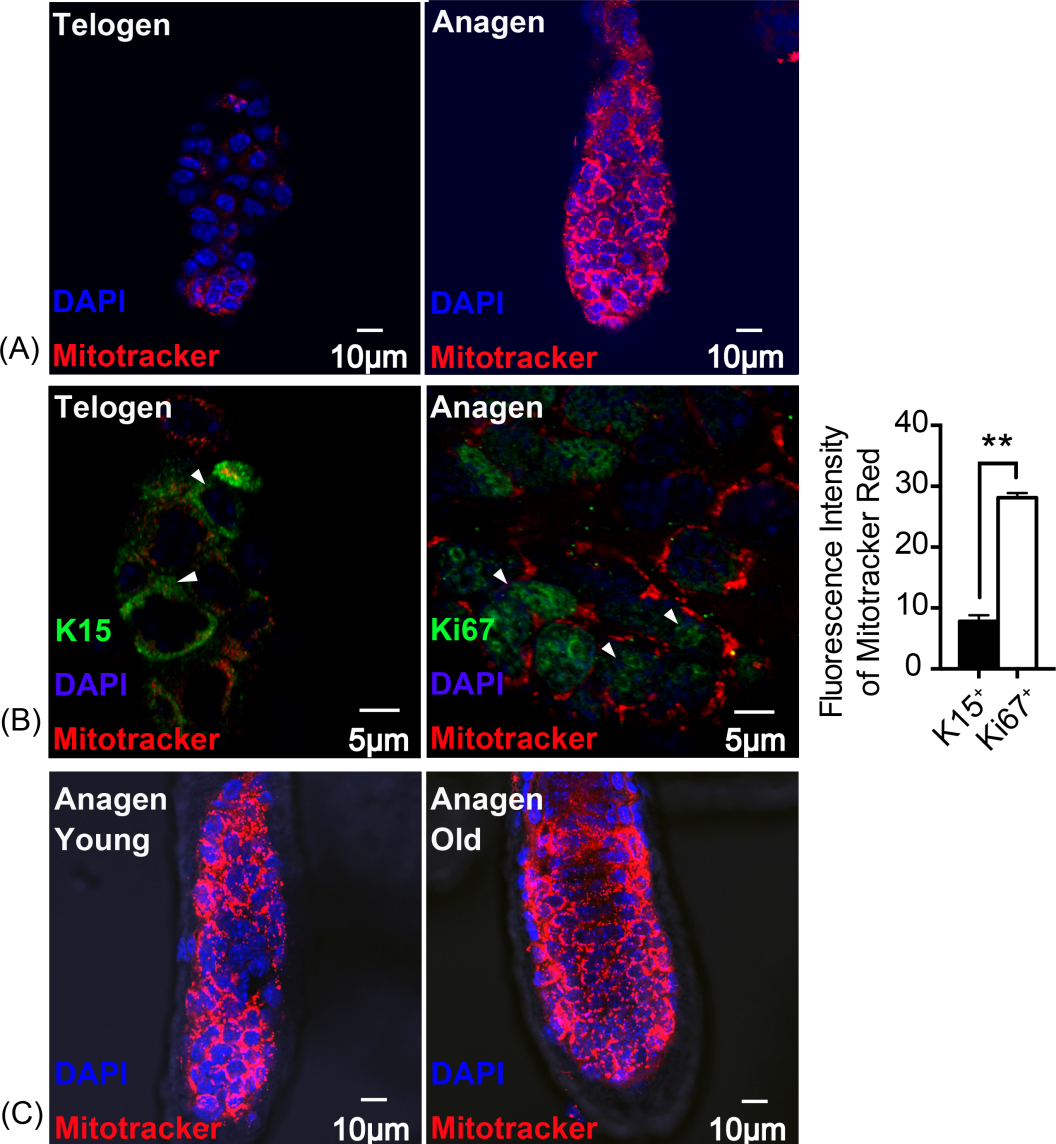
Mitochondrial activity is increased in anagen proliferating matrix cells. Fluorescence intensity of Mitotracker Red was detected to measure mitochondrial activity. (A) More mitochondria and a higher mitochondrial activity were detected in anagen matrix cells than in telogen bulge cells. (B) Mitochondrial activity was significantly elevated in Ki67^+^ proliferating cells than in K15^+^ stem cells (^∗∗^, *P* < 0.01). (Markers flagged by white arrows) (C) Mitochondria in matrix cells were of same activity levels due to in different aged mice. (A, B) Data show a complication of 3 experiments (*n* = 3 mice per group, with two 5 mm × 5 mm sections per mouse). (C) Data show a complication of 3 experiments (*n* = three 8 week-old mice (young group) and three 2 year-old mice (old group) per experiment, with two 5 mm × 5 mm sections per mouse).

Thus, mitochondrial activity was assessed using Mito Tracker Red. The result indicates that, fluorescence intensity was significantly increased in anagen matrix cells compared with telogen bulge cells (Fig. 2A). Keratin 15 (K15) is known as a marker for stem cells while Ki67 symbolizes proliferating matrix cells (Eisinger et al., 2010). To locate HFSCs and proliferating HF matrix cells precisely, K15 and Ki67 were detected as shown in Fig. 2B. And there is an approximately three times increase in the fluorescence intensity of Mitotracker Red in Ki67^+^ proliferating cells than that of K15^+^ stem cells (Fig. 2B), suggesting an enhancement in mitochondrial activity during HFSCs differentiation, which is in accordance with the feature of embryonic stem cells (Chung et al., 2007). In addition, mitochondrial activity in young mice (8 week-old) does not differ from that of the old mice (2 year-old) (Fig. 2C), indicating that ageing does not have an significant influence on mitochondrial activity during stem cell differentiation.

### Redox balance was sustained through enhancing SOD2 expression

**Figure 3:**
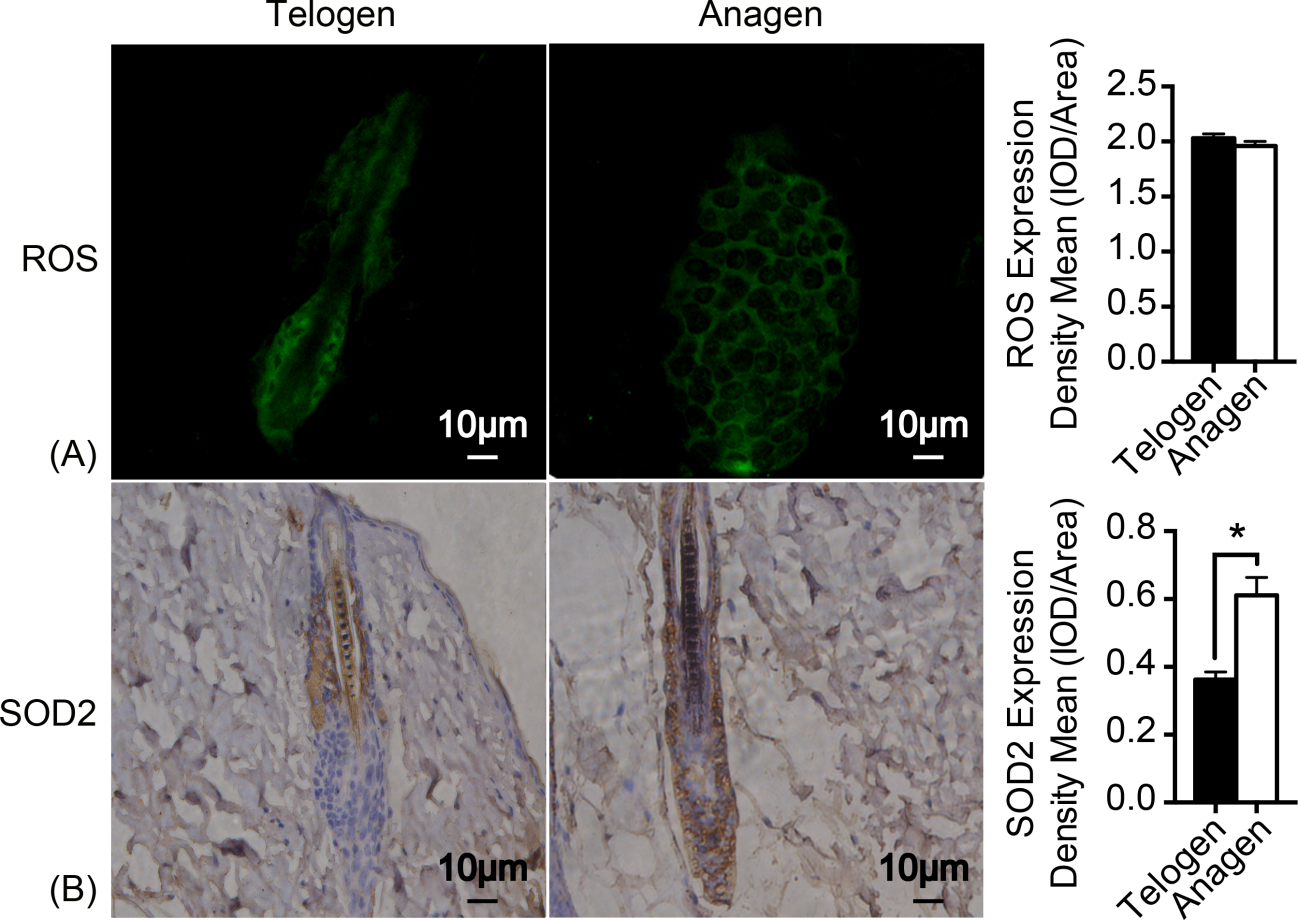
SOD2 is increased in anagen matrix cells to maintain redox homeostasis. (A) There was no significant difference in ROS expression between telogen bulge cells and anagen matrix cells (*P* > 0.05). (B) SOD2 expression was significantly enhanced in anagen matrix cells (^∗^, *P* < 0.05). Data show a complication of 3 experiments (*n* = 3 mice per group, with two 5 mm × 5 mm sections per mouse).

As mitochondrion are the major generator of endogenous ROS in cells and electrons that leak out from the electron transport chain contribute to the production of ROS (Chen et al., 2008). H2DCFDA (2′, 7′-dichlorodihydrofluorescein diacetate) immunofluorescence was used to measure ROS levels in HFSCs. Unexpectedly, ROS levels are almost identical in these two stages of cell types (Fig. 3A). Nonetheless, expression of respiratory enzyme SOD2 was significantly improved in anagen matrix cells compared with that in the telogen bulge cells (Fig. 3B). We speculate that SOD2 levels are upregulated in anagen matrix cells to clear ROS during the differentiation process for redox homeostasis. Expression of SOD1, another respiratory enzyme, was also estimated during HFSC differentiation, but showed no significant difference (data not shown).

### Change of respiratory enzymes expression during HFSCs differentiation

**Figure 4:**
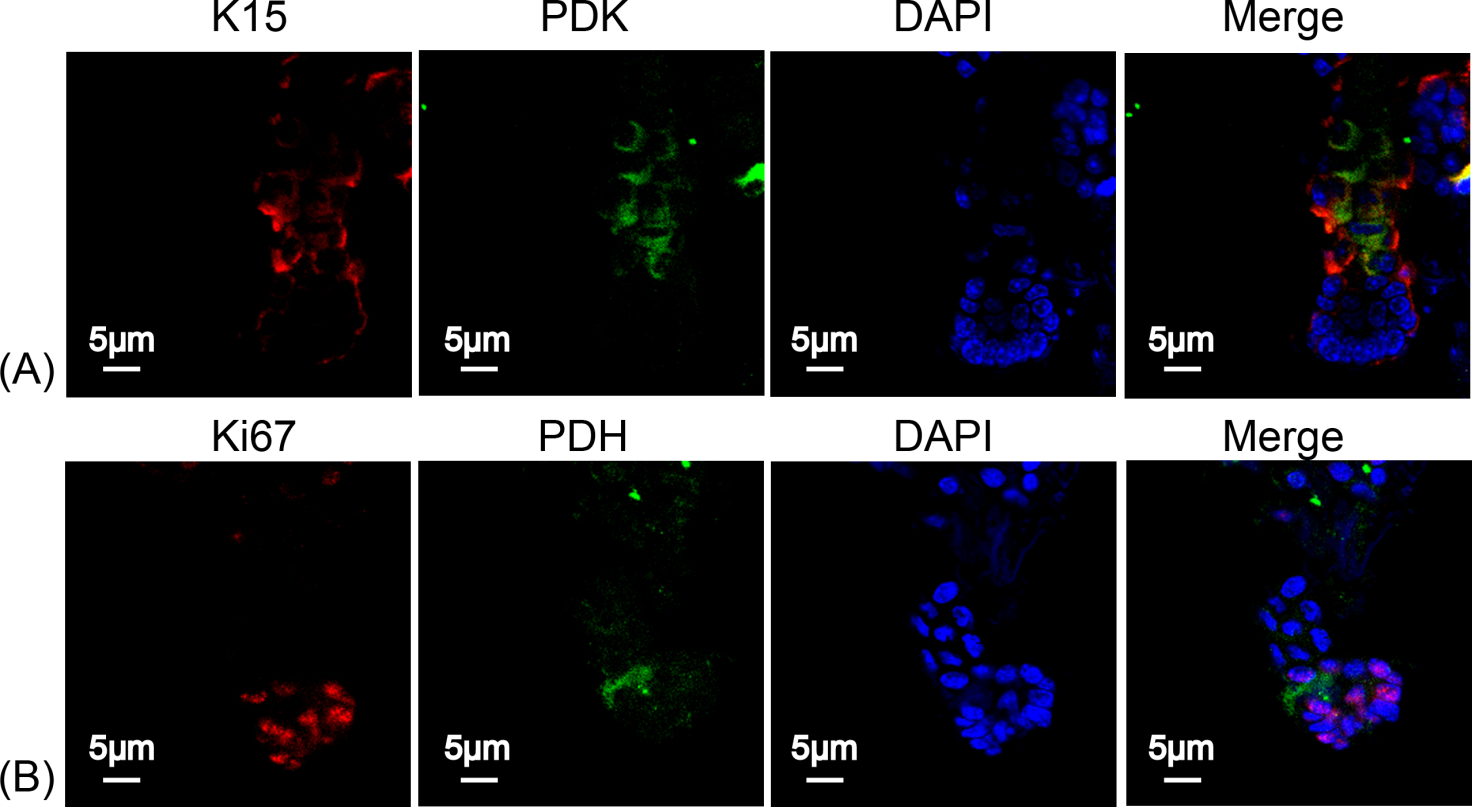
HFSCs present anaerobic respiration, while proliferating matrix cells show oxidative phosphorylation. Immunofluorescence detection of PDK and PDH during HFSC differentiation. (A) PDK is mainly expressed in K15^+^ stem cells. (B) PDH is mainly expressed in Ki67^+^ proliferating cells. Data show a complication of 3 experiments (*n* = 5 mice per group, with two 5 mm × 5 mm sections per mouse).

To determine the type of metabolism used by HFSCs, we again measured respiratory enzymes in K15^+^ stem cells and in Ki67^+^ proliferating cells. As shown in Fig. 4, PDK was highly expressed in HFSCs (Figs. 4A) while PDH was highly expressed in differentiated cells (Fig. 4B), indicating that anaerobic mitochondrial metabolism plays a dominant role in HFSCs, whereas aerobic metabolism is essential in differentiated cells.

### Suppression of mitochondrial oxidative phosphorylation delays hair regeneration

**Figure 5:**
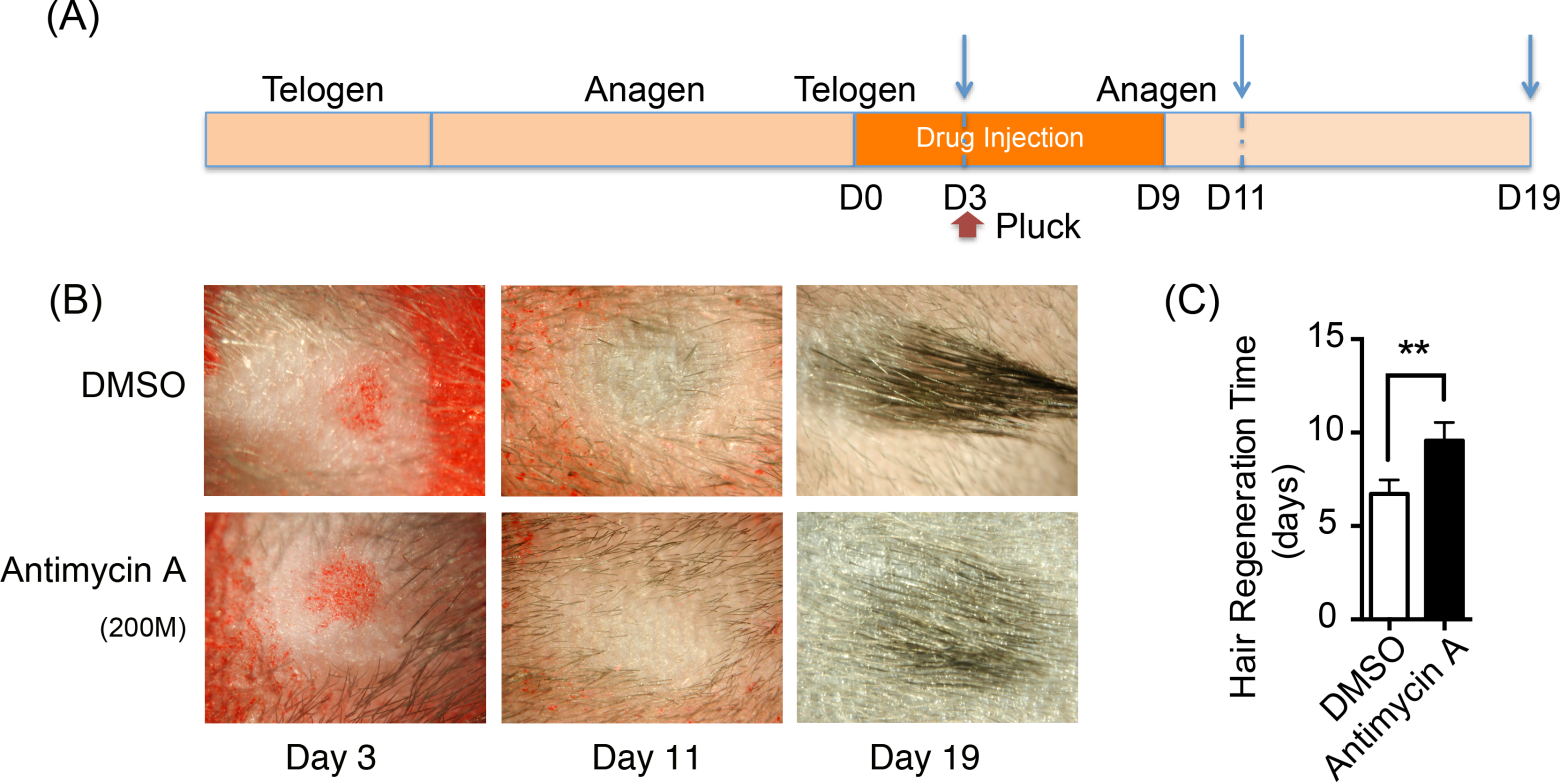
Inhibition of mitochondrial respiration retards hair regrowth. (A) Schematic diagram of our experimental approach. Mice were treated with Antimycin A intracutaneously on one side of the back skin, and DMSO on the contralateral side for 10 days. 200 hairs were plucked after three days of drug treatment (as shown in red arrow). Photos were taken at day 3, day 11 and day 19 after the start of treatment (as shown in blue arrow). (B) Photos of hair regrowth taken at day 3, 11, and 19 after treatment. At day 11, hair growth was observed in the control group (treated with DMSO) while hairs failed to grow in the antimycin A group, indicating that hair regeneration was suppressed in the antimycin A group. (C) It took much longer time in the antimycin A group (9.6 ± 0.9 days) in hair regrowth than that in the control group (6.7 ± 0.7 days) (^∗∗^, *P* < 0.01). Data show a complication of 3 experiments (*n* = 3 mice per group).

Oxidative phosphorylation increases during HFSCs differentiation, which is supplied mainly via the respiratory pathway (Armstrong et al., 2010). It is temping to speculate that disrupting mitochondrial oxidative phosphorylation might inhibit the differentiation and proliferation of hair stem cells and retard hair regeneration. Hence, a respiratory inhibitor, antimycin A [complex III inhibitor], was injected on one side of mouse dorsal skin subcutaneously to prohibit mitochondrial activity. DMSO was treated on the contralateral side as the control. The treatment process is summarized in Fig. 5A. 200 hairs were plucked after three days of drug treatment. After plucking, hair regrowth was recorded in these treated regions and the appearance of neonatal hair was monitored by taking photographs each day (Fig. 5B). The antimycin A group showed significant delays (9.6 ± 0.9 days) in hair growth compared with the control group (6.7 ± 0.7 days), as shown in Fig. 5C (*P* < 0.05). Accordingly, disruption of mitochondrial respiration leads to the delay of hair follicle regrowth, revealing that alteration of mitochondrial respiratory function might be essential in HFSCs differentiation.

## Discussion

Emerging studies focus on the effect of mitochondria in regulating stem/progenitor cell differentiation and proliferation. For instance, mitochondrial ROS signal transduction was found of importance in regulating keratinocyte differentiation (Hamanaka & Chandel, 2013; Hamanaka et al., 2013). Also, mitochondria negatively regulate proliferation and differentiation of embryonic mouse cerebral cortical neural progenitor cells (NPCs) through generating superoxide (Yan Hou et al., 2012). Differentiation of bmMSCs was accompanied by distinct regulation of mitochondrial bioenergetics, providing a novel way in manipulating cell fate of MSCs (Shum et al., 2016). Crucially, deletion of mitochondrial transcription factor A (Tfam(EKO)), which induces loss of the electron transport chain (ETC) in epidermis, restrains entire skin development, including hair follicle differentiation and proliferation (Kloepper et al., 2015). Despite the importance of mitochondria in regulating cell differentiation, the alterations of mitochondrial morphology and its respiratory function during HFSCs differentiation are poorly understood. Hence, we revealed in this paper that the change in mitochondrial morphology and activity, redox homeostasis and metabolic bioenergetics of HFSCs during differentiation.

Mitochondria display cycles of fission and fusion, showing a dynamic morphology and function changes (Willems et al., 2015). Differentiated hair follicle cells showed more sophisticated mitochondrial ultrastructure with elongated shape (*P* < 0.01) and more cristae protruding into the matrix compared to HFSCs (Fig. 1). Simultaneously, differentiated hair follicle cells showed higher mitochondrial activity based on the fluorescence intensity of Mitotracker Red (*P* < 0.05) (Fig. 2A). It has been suggested that K15 and Ki67 are biomarkers for epidermal stem cells and proliferating cells, respectively (Bose et al., 2013; Ohta Y, 2000; Scholzen T, 2000). We used these markers for accurate location of HFSCs and its differentiated counterparts. Again, the result confirmed the phenomenon as described above that mitochondrial are more active in differentiated hair follicle cells (Fig. 2B). It has been revealed previously that mitochondria became elongated ones with swollen cristae in differentiated ESCs to prepare for aerobic metabolism (Facucho-Oliveira & St John, 2009). Additionally, mitochondria increased and became more sophisticated in ultrastructure as described above in differentiation of human MSCs and human ESCs (Chen et al., 2008; Cho et al., 2006). Even in female primordial germ cell, mitochondria transform from rounded with small vesicular cristae into elongated one with parallel, arched cristae upon differentiation (Pietro M Motta, Sayoko & Rosemarie, 2000). Hence, it is assumed that mitochondrial ultrastructure and activity altered to adapt to the demand of energy supply during HFSCs differentiation.

ROS, a principle production of mitochondrial metabolism, regulates the redox balance along with respiratory enzymes, such as SOD2. Also, ROS was discovered as a secondary signal pathway in regulating cell differentiation, such as keratinocytes and neural progenitor cells. It has been reported that ROS promotes cell senescence, such as bmMSCs, as well (Hamanaka et al., 2013; Wu et al., 2014; Yan Hou et al., 2012). However, ROS were not significantly altered upon HFSCs differentiation, showing no difference between telogen bulge stem cells and anagen differentiated cells (Fig. 3A), though mitochondrial activity was distinctly increased. To better present redox status, SOD1 and SOD2 were also measured. Expression of SOD2 was upregulated during HFSCs differentiation (Fig. 3B), but SOD1 was not (data not shown). Elevated SOD2 expression during differentiation of iPSCs and neuroblastoma cells was discovered in previous studies for sustaining redox homeostasis and preventing ROS-induced cell death (Armstrong et al., 2010; Case et al., 2013; Ruggeri et al., 2014). Interestingly, enhanced mitochondrial activity upon HFSCs differentiation is age-independent (Fig. 2C), while deletion of SOD2 results in diverse effect of mitochondrial dysfunction on epidermal stem cells in young and old mice (Michael C Velarde, Simon & Judith, 2015). In addition, overexpression of SOD2 was proved to be protective in myoblast mitochondrial mass and function during ageing (Lee, Van Remmen & Csete, 2009), indicating that mitochondrial activity and function might be protected by SOD2 expression during ageing according to our result. These results collectively suggested that SOD2 plays an essential role in maintaining the redox homeostasis.

Except for redox homeostasis, mitochondrial metabolic function is of great importance in regulating hair growth. PDH is responsible for the conversion of pyruvate into acetyl CoA to enter the tricarboxylic acid cycle and aerobic metabolism representing aerobic respiration. Besides, PDK inhibits the activity of PDH by phosphorylation, to enhance anaerobic respiration. Therefore, PDH and PDK were measured in HFSCs and differentiated HF cells to determine the respiration rate. Our results indicated that anaerobic glycolysis is mainly conducted in HFSCs, while oxidative phosphorylation in differentiated cells, revealing an anaerobic to aerobic transition pattern (Fig. 4). Similarly, oxidative phosphorylation was activated in MSCs during osteogenic differentiation (Shum et al., 2016). To explore the significance of alteration in mitochondrial energetic metabolism during differentiation, antimycin A was used *in vivo* followed by hair plucking at the third day. The results revealed that disrupting mitochondrial respiration delays hair regrowth after plucking (Fig. 5). It is possible that hair regeneration might be retarded due to insufficient energy supply. Another possibility is that mitochondrial dysfunction affects HFSCs differentiation through regulating redox balance or other signaling pathways, leading to the delay of hair growth. Mitochondria exert pleiotropic effects on cell differentiation through different signaling pathways. For instance, down-regulation of DRP suppresses Notch and subsequently suppressing follicle cell differentiation in *Drosophilia*. ROS inhibits epidermal differentiation through decreasing Notch signaling. Furthermore, inhibiting nuclear translocation of apoptosis-inducing factor (AIF), which was released from mitochondria, retards anagen-to-catagen phase transition of hair follicle growth cycle and leads to decreased hair regeneration (Lan et al., 2015). Further research is needed to reveal if mitochondrial metabolic dysfunction inhibits hair regeneration through regulating HFSCs cell differentiation and its signaling pathway.

## Conclusion

In summary, mitochondria are elongated with parallel, arched cristae with higher activity in differentiated hair follicle cells. Increased SOD2 is capable of maintaining redox homeostasis, preventing from ROS induced injury. In addition, HFSCs present anaerobic glycolysis, while switch to mitochondrial oxidative phosphorylation after differentiation. And inhibiting mitochondrial metabolic function retards hair regeneration.

## Supplemental Information

10.7717/peerj.1821/supp-1 Supplemental Information 1 Raw data for Fig. 1

10.7717/peerj.1821/supp-2 Supplemental Information 2 Raw data for Fig. 2

10.7717/peerj.1821/supp-3 Supplemental Information 3 Raw data for Fig. 3

10.7717/peerj.1821/supp-4 Supplemental Information 4 Raw data for Fig. 4

10.7717/peerj.1821/supp-5 Supplemental Information 5 Raw data for Fig. 5

## Additional Information and Declarations

### Data Availability

The following information was supplied regarding data availability: Raw data is available as Supplemental Information.
