# Supplementary figures and images for: Mitochondrial aerobic respiration is activated during hair follicle stem cell differentiation, and its dysfunction retards hair regeneration
Source: PeerJ. 2016 May 3;4:e1821. doi: 10.7717/peerj.1821 (PMC4860312; doi:10.7717/peerj.1821)

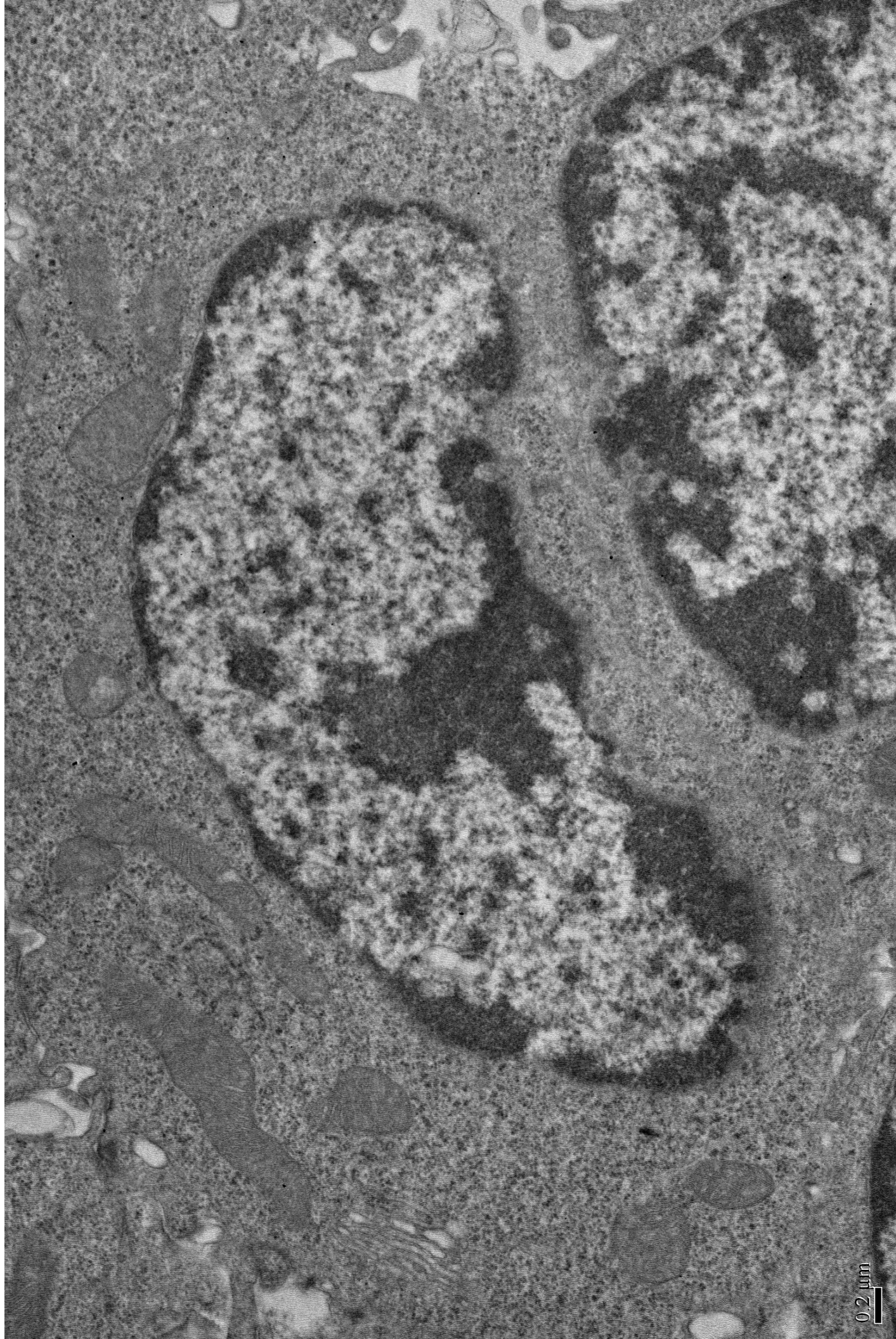

0.2  $\mu$ m

Supplement: Supplemental Information 1 [file peerj-04-1821-s001.pdf]

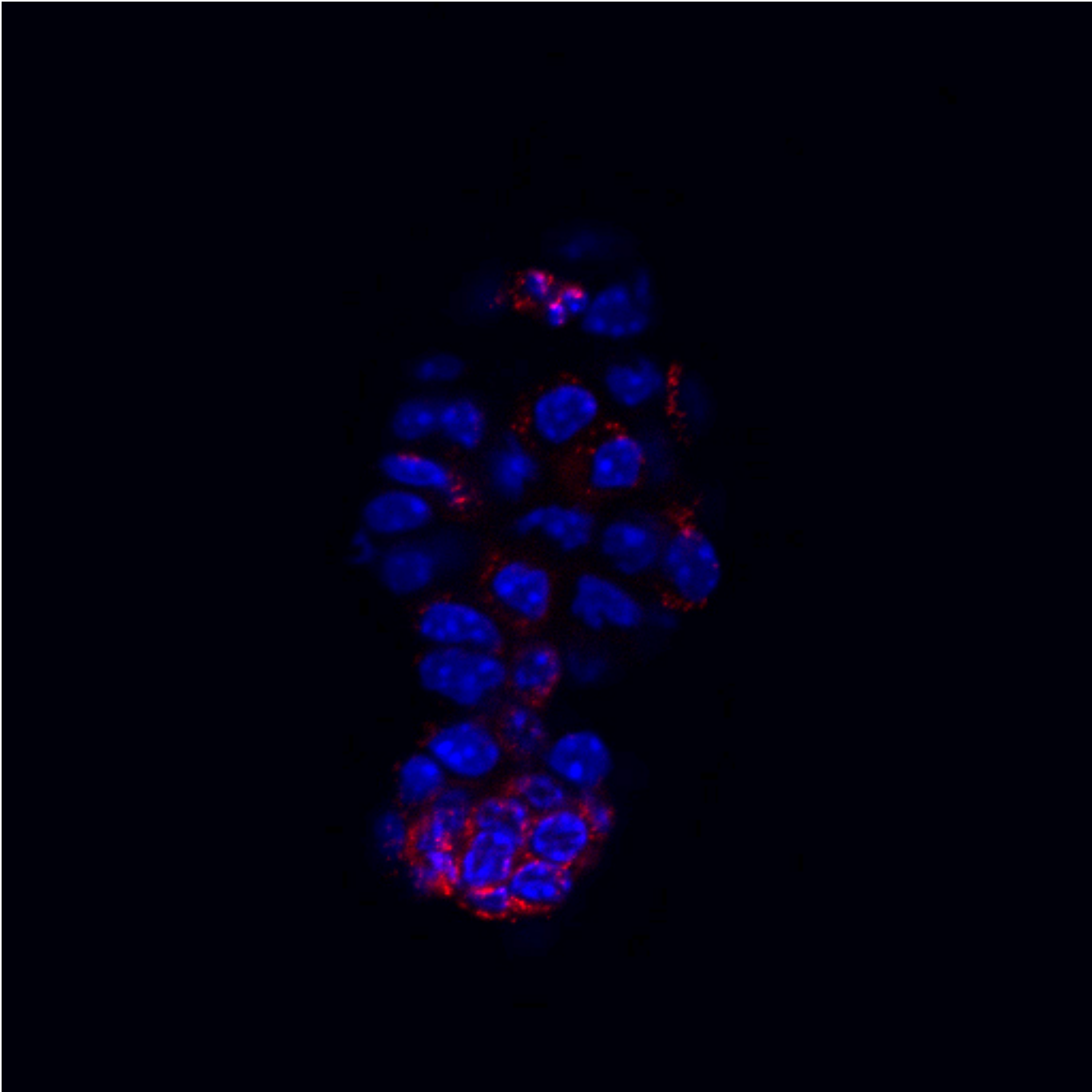

Supplement: Supplemental Information 2 [file peerj-04-1821-s002.zip › Raw data or figure 2/peerj-8406-Fig_2-a1.pdf]

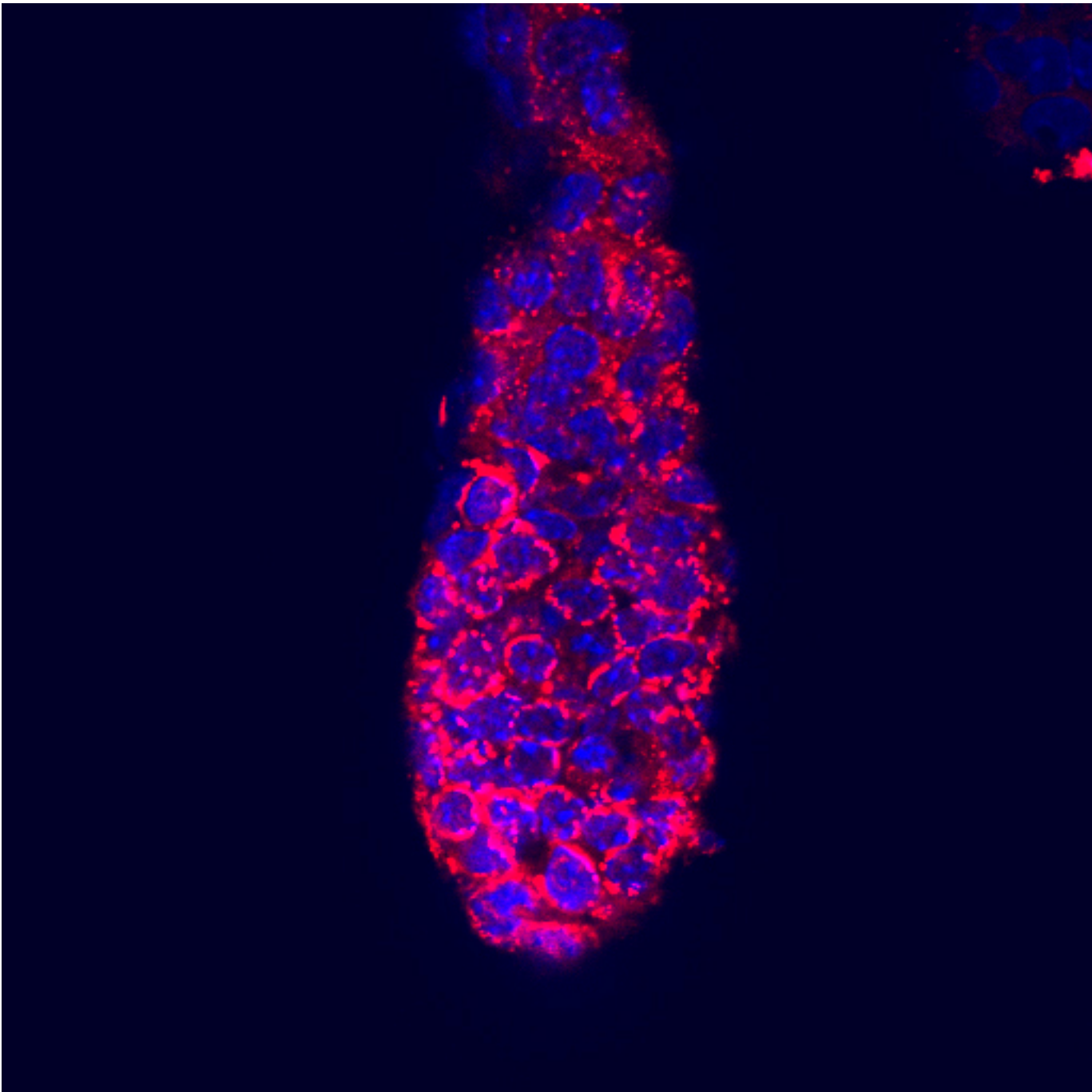

Supplement: Supplemental Information 2 [file peerj-04-1821-s002.zip › Raw data or figure 2/peerj-8406-Fig_2-a2.pdf]

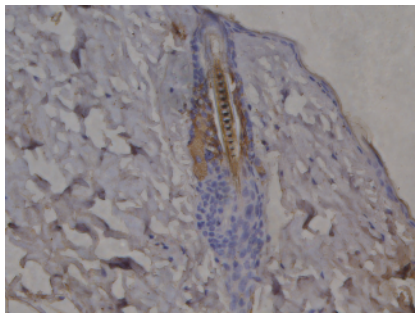

Supplement: Supplemental Information 3 [file peerj-04-1821-s003.pdf]

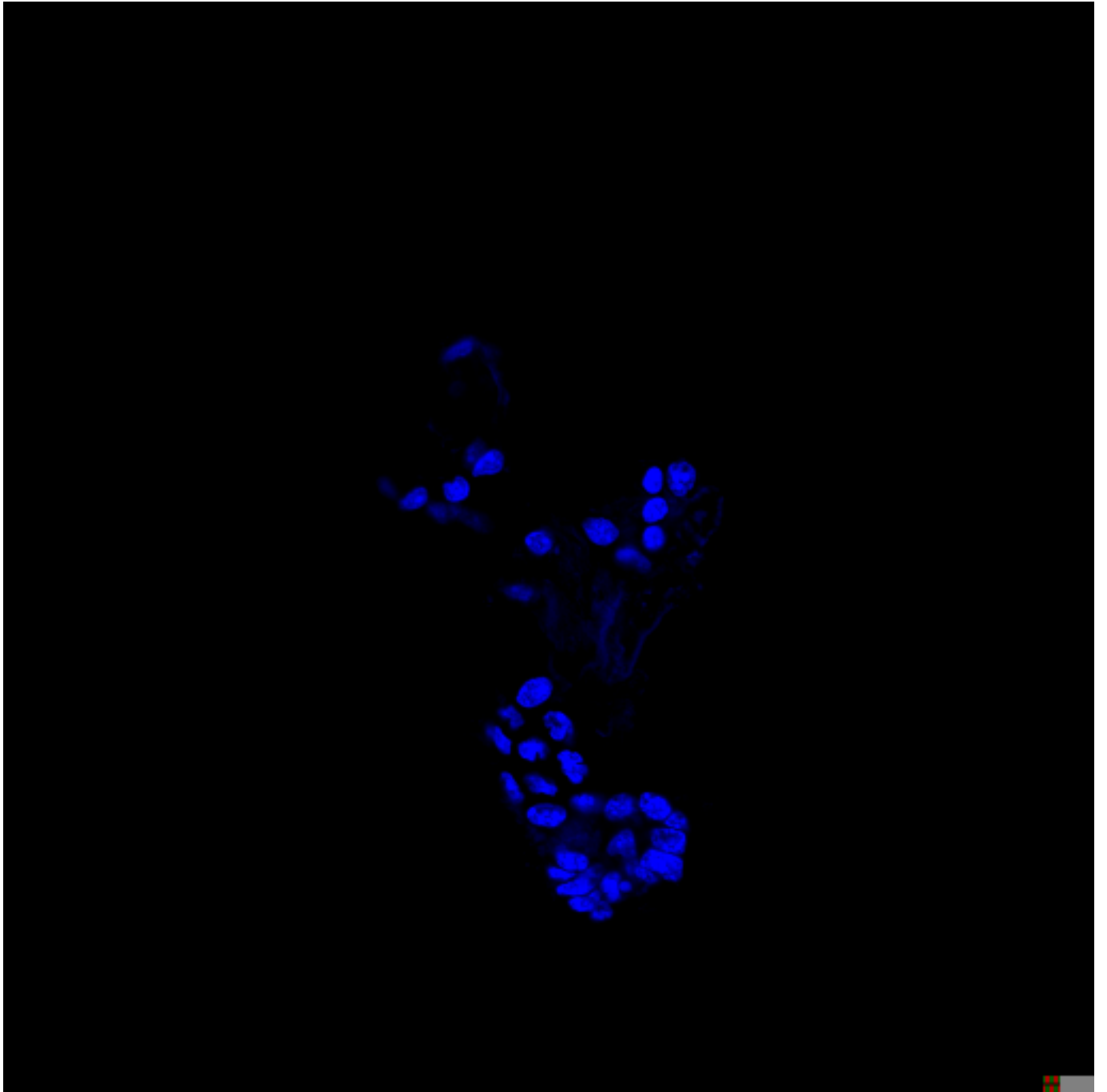

Supplement: Supplemental Information 4 [file peerj-04-1821-s004.pdf]

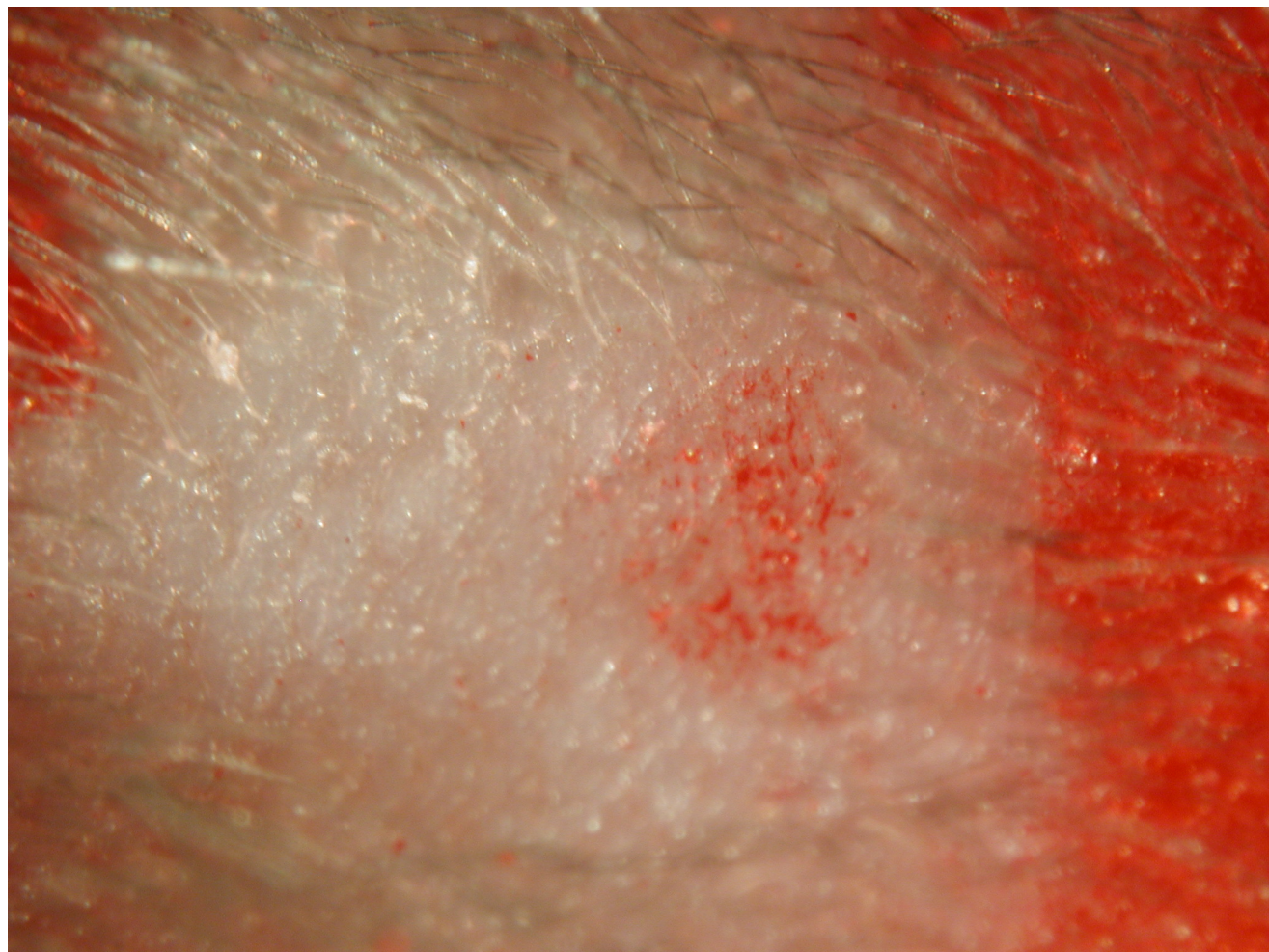

Supplement: Supplemental Information 5 [file peerj-04-1821-s005.pdf]
